# Mini-Review: Nod Factor Regulation of Phytohormone Signaling and Homeostasis During Rhizobia-Legume Symbiosis

**DOI:** 10.3389/fpls.2018.01247

**Published:** 2018-09-03

**Authors:** William P. Buhian, Sandra Bensmihen

**Affiliations:** LIPM, Université de Toulouse, INRA, CNRS, Toulouse, France

**Keywords:** auxin, cytokinins, gibberellin, ethylene, strigolactone, infection, nodule organogenesis, LCOs

## Abstract

The rhizobia-legume symbiosis is a mutualistic association in which bacteria provide plants with nitrogen compounds and the plant provides bacteria with carbon sources. A successful symbiotic interaction relies on a molecular dialog between the plant and the bacteria, and generally involves rhizobial lipo-chitooligosaccharide signals called Nod factors (NFs). In most cases, specific NF perception is required for rhizobia to enter root cells through newly formed intracellular structures called infection threads (ITs). Concomitantly to IT formation in root hairs, root cortical cells start to divide to create a new root organ called the nodule, which will provide the bacteria with a specific micro-environment required for symbiotic nitrogen fixation. During all these steps of plant–bacteria interaction, new plant cellular compartments and developmental programs are activated. This interaction is costly for the plant that tightly controls symbiosis establishment and functioning. Phytohormones are key regulators of cellular and developmental plasticity in plants, and they are influential endogenous signals that rapidly control plant responses. Although early symbiotic responses were known for decades to be linked to phytohormone-related responses, new data reveal the molecular mechanisms involved and links between phytohormones and the control of early symbiotic events. Reciprocally, NF signaling also targets phytohormone signaling pathways. In this review, we will focus on the emerging notion of NF and phytohormone signaling crosstalk, and how it could contribute to the tight control of symbiosis establishment in legume host plants.

## Introduction

Legume plants can interact with soil bacteria, named rhizobia, to establish the rhizobium legume (RL) symbiosis. Legumes host Rhizobia in specific root organs called nodules, where they fix atmospheric nitrogen. This symbiosis provides the plant with nitrogen compounds (ammonium) and the plant provides the bacteria with carbon sources. The efficiency of this interaction relies for a great part on the massive intracellular infection of plant cells by rhizobia and the protective structure of the nodule. The plant host must invest a great deal of energy to both house and support nitrogen fixing bacteria, and as such, must tightly regulate this process.

Nod factors (NFs) are lipo-chitooligosaccharide (LCO) molecules produced by rhizobia in response to flavonoids present in root exudates. NFs are generally essential for the onset and the maintenance of the RL symbiosis (Dénarié, 1996). Intriguingly, NFs can also stimulate lateral root formation via the symbiotic signaling pathway implying that similar host mechanisms are involved in both nodule and lateral root development (Olah et al., 2005). Genetic pathways governing NF perception and signaling are now quite well understood (Gough and Cullimore, 2011). NFs are perceived by the plant at the root epidermis and are likely produced by rhizobia all along the infection process (Sharma and Signer, 1990).

Rhizobia first attach to root hairs (RHs) in a susceptible zone of host roots (Bhuvaneswari et al., 1981) and then enter RHs through a membrane invagination called an infection thread (IT). Even before the IT is formed, pericycle cells are activated and divide to start organogenesis of the nodule (Timmers et al., 1999). NFs are usually essential for the infection process and, in *Medicago sativa*, purified NFs can trigger pericycle activation and initiate nodule organogenesis (Truchet et al., 1991; Timmers et al., 1999). Defects in the infection process often result in defective nodule organogenesis. Infection and organogenesis events of the RL symbiosis are thus tightly coordinated. This tight coordination and signaling across root layers, and the events of cellular reactivation and cell division associated with symbiotic establishment strongly imply the involvement of hormonal pathways.

Recent transcriptomic data suggest that NF signaling regulates the host hormone biosynthesis and signal transduction pathways. Hormones function in the pico to nano-molar range (Gaspar et al., 2003). Their activity depends on a tight balance of activation (biosynthesis) and inactivation (conjugation, degradation) pathways, as well as transport and signaling. In this mini-review, we will focus on data showing positive and negative feedback regulatory mechanisms between NF signaling and hormonal pathways. We present data from legumes producing both indeterminate (*Medicago truncatula*, pea) and determinate [*Lotus japonicus*, *Glycine max* (soybean)] nodules. These two types of nodule differ in the site of initial cortical divisions, persistency of the nodule meristem and auxin sensitivity (Bensmihen, 2015; Ng and Mathesius, 2018). Effects of NF signaling on both hormone homeostasis and hormone signaling will be discussed.

## NF Signaling and Regulation of Hormone Homeostasis

**FIGURE 1:**
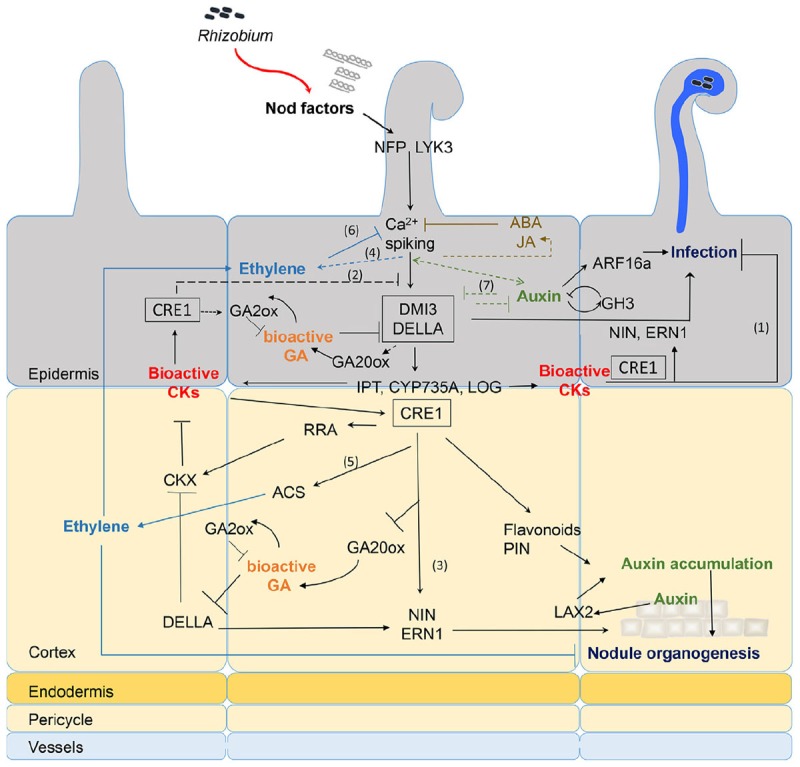
Recapitulative spatio-temporal scheme of the interactions between NF signaling and downstream hormonal pathways. Rhizobia produce NFs that are perceived in root hairs (RHs) through LysM-RLK receptors such as NFP and LYK3. NF perception leads to calcium spiking and activation of DMI3 that acts upstream of the CK receptor MtCRE1. The signaling cascade involving DMI3 and CRE1 is likely involved in both epidermal and cortical signaling (black boxes). NF treatment triggers early CK biosynthesis gene (*IPT/CYP735A/LOG*) expression and CK accumulation in *M. truncatula* roots downstream of *DMI3* but independently of CRE1 signaling (van Zeijl et al., 2015b). Although tissue specificity of this CK production was not determined, evidence from *L. japonicus* and *M. truncatula* suggests that epidermal CK accumulation is a negative regulator of RH infection (1) and NF signaling (2) (Held et al., 2014; Jardinaud et al., 2016). In contrast, cortical CK is a positive regulator of nodule organogenesis (3) (Gonzalez-Rizzo et al., 2006; Murray et al., 2007; Reid et al., 2017). Bioactive CKs are perceived by CRE1 and induce expression of *NIN* and *ERN1* (Ariel et al., 2012), which are positive regulators of both infection and nodule organogenesis (Andriankaja et al., 2007; Marsh et al., 2007). This induction might be partly through regulation of DELLA activities. GA is a negative regulator of DELLA protein stability. Bioactive GA pools are likely present in both epidermis and cortex early after NF treatment (Fonouni-Farde et al., 2016; Jardinaud et al., 2016; Herrbach et al., 2017). DELLAs play a positive regulatory role on symbiotic gene expression such as *ERN1* and they negatively regulate CK degradation (Fonouni-Farde et al., 2016, 2017; Jin et al., 2016). In contrast, CK positively regulate GA inactivation enzymes and down-regulate expression of the GA20ox activation enzyme (Fonouni-Farde et al., 2018), suggesting a negative feedback of CK on GA active pools. NF signaling induces ethylene production, both independently of the LHK1/CRE1 CK pathway (4) (Reid et al., 2018) and downstream of CK perception (5) (van Zeijl et al., 2015b). Ethylene reduces CK production in *M. truncatula* roots, possibly through negative feedback on NF signaling (6) (van Zeijl et al., 2015b). Ethylene negatively regulates NF-induced calcium spiking, RH infection, and nodule organogenesis (Heidstra et al., 1997; Penmetsa et al., 2008). Regulation of auxin biosynthetic and conjugation enzyme (GH3) genes occurs in NF treated RHs and upon *S. meliloti* inoculation in an NF-dependent manner (Breakspear et al., 2014; Larrainzar et al., 2015; Jardinaud et al., 2016; Herrbach et al., 2017). Reciprocal positive and negative feedback regulatory loops between some auxin and NF regulated genes (7) was shown by comparing the combined effect of auxin and NFs to either treatment alone (Herrbach et al., 2017). Downstream of CK perception, control of auxin transport in the cortex seems regulated by differential expression of *PIN* genes (Plet et al., 2011) or accumulation of flavonoid compounds. Auxin accumulation is also mediated by *MtLAX2* that is induced upon *S. meliloti* infection in vasculature and early nodule primordia, and which is required for nodule organogenesis (Roy et al., 2017). In parallel, epidermal auxin signaling controls infection thread (IT) formation, at least partly through *ARF16a* (Breakspear et al., 2014). Exogenous application of high concentrations (μM range) of ABA or JA inhibits NF-induced calcium spiking (Sun et al., 2006; Ding et al., 2008) but this inhibition is so far not supported by any transcriptomic data. Plain bars represent negative and plain arrows positive regulations. Dashed lines are hypothetical relationships, and solid lines have evidence from the literature. Different hormones are highlighted by different colors. CK, cytokinins; GA, gibberellins; ABA, abscisic acid; JA, jasmonic acid; NF, Nod factors. Bacteria entrapped in curled root hair and IT are shown in blue.

Phytohormones are key regulators of plant growth and responses to biotic and abiotic stresses. Several transcriptomic studies show that hormone biosynthesis, activation or degradation genes are differentially expressed upon NF treatment (see **Table 1** and **Figure 1**).

**Table 1:** Summary of major hormone homeostasis and signaling genes differentially regulated by Nod factors or rhizobia during symbiosis.

		Role	Regulation	Representative functional studies, if present
Auxin	*TAR*	Auxin biosynthesis	UP (10 h NF+ NAA^9^)	
	*YUCCA, ASA*	Auxin biosynthesis	UP (>0.5 h^6^)	
	*ARF16a*	Auxin signaling	UP (24 h)^2^	Controls infection at earliest stages. *ARF16a* mutants show a decreased number of infection events^2^.
	*GH3.1*	Auxin conjugation	UP (4 h^1^, 24 h^2^)	Found to be enriched in nodules, and has distinct expression patterns, activated first in epidermis than in cortex upon *S. meliloti* infection^5^. GmGH3-14, 15 control nodule number and size^3^.
	*PIN2, PIN4, PIN10*	Polar auxin transport	Indirect control through MtCRE1^4,5^	Differential expression dependent on CRE1^4^. Application of auxin transport inhibitors rescues *Mtcre1* phenotype^5^.
	*LBD18, LBD16*	Auxin signaling	UP (6 hpi)^a,6^	
	*PLT*	Auxin signaling	UP (6 hpi^a,6^, 4 h^1^)	Required for the maintenance of the nodule meristem (NM). Downregulation of all MtPLT produced nodules deficient in NM and infection zone^8^.
CK	*IPT*	Cytokinin biosynthesis	UP (3 h^7^, 4 h^1^, 10 h^9^, 24 h^1^)	Early response is not LHK1 dependent. Overexpression of the entire biosynthetic pathway is necessary for visible phenotypes^12^.
	*CYP735A, APT4*	Cytokinin biosynthesis	UP (3 h^7^, 4 h, 24 h^1^)	
	*LOG1, LOG2*	Cytokinin biosynthesis	UP (3 h)^7^	Expression in RL symbiosis dependent on *MtCRE1*^7^. *MtLOG1* positively regulates nodule organogenesis, and in autoregulation of nodulation^13^.
	*MtCRE1/LjLHK1*	Cytokinin signaling	UP (4 h^1^)	*Ljhit1/lhk1* mutants are hyperinfected, infection threads (ITs) fail to penetrate cortex ^10^. *MtCRE1* controls IT progression and nodule organogenesis^11^.
	*RR4, RR5, RR8, RR9, RR11*	Cytokinin signaling	UP (3 h^16^, 4 h^1^) UP (48 hpi)^a,15^	Spatiotemporal expression as function of Nod factor response of (RR9, RR11) largely overlap. Controls lateral root and nodule number^16^.
	*CKX*	Cytokinin catabolism	UP (3 h^7^)	Expressed in nodulation. Mutants show decreased infection and nodulation efficiency^14^. Synergistic NF+auxin regulation^9^.
Ethylene	*ACS, ACS3*	Ethylene biosynthesis	UP (3 h)^6,7^	
	*ACO*	Ethylene biosynthesis	UP (1 h^19^, 10 h NF + NAA^9^, 0.5–3 h^6^)	
	*EIN2/MtSKL*	Ethylene signaling	Down (10 h NF + NAA^9^)	EIN2 regulates IT growth and cortical cell division; also regulates number and distribution of infection events^17,18^.
GA	*Ga20ox*	GA biosynthesis	UP (3 h^7^, 24 h^1^)	
	*Ga2ox*	GA catabolism	UP (24 h^2^)	
SL	*D27*	SL biosynthesis	UP (3 h^20^, 24 h^1,2^)	Nod factor-induced expression dependent on NSP1 and NSP2^20^
	*CCD8*	SL biosynthesis	UP (5 dpi^a,2^)	
JA	*AOS*	JA biosynthesis	DOWN (24 h^2^)	
	*JAZ, COI*	JA signaling	DOWN (24 h^2^)	
BR	*HYD1*	BR synthesis	DOWN (48 hpi)^a,15^	
	*DWARF1*	BR synthesis	DOWN (5 dpi)^a,2^	


*Data from Breakspear et al. (2014) and Jardinaud et al. (2016) were obtained from epidermal samples. All other transcriptomic data were obtained from whole root samples of *Medicago truncatula* (Larrainzar et al., 2015; van Zeijl et al., 2015b; Herrbach et al., 2017), *Glycine max* (Hayashi et al., 2012), and *Lotus japonicus* (Miyata et al., 2013). Unless otherwise indicated, samples were treated with Nod factors. ^*a*^Root samples inoculated with rhizobia. CK, cytokinins; GA, Gibberellins; SL, strigolactones; JA, jasmonic acid; BR, brassinosteroids; NF, Nod factors. ^*1*^Jardinaud et al., 2016. ^*2*^Breakspear et al., 2014. ^*3*^Damodaran et al., 2017. ^*4*^Plet et al., 2011. ^*5*^Ng et al., 2015. ^*6*^Larrainzar et al., 2015. ^*7*^van Zeijl et al., 2015b. ^*8*^Franssen et al., 2015. ^*9*^Herrbach et al., 2017. ^*10*^Murray et al., 2007. ^*11*^Gonzalez-Rizzo et al., 2006. ^*12*^Reid et al., 2017. ^*13*^Mortier et al., 2015. ^*14*^Reid et al., 2016. ^*15*^Hayashi et al., 2012. ^*16*^Op den Camp et al., 2011. ^*17*^Penmetsa and Cook, 1997. ^*18*^Penmetsa et al., 2003. ^*19*^Miyata et al., 2013. ^*20*^van Zeijl et al., 2015a.*

### Cytokinins

#### Biosynthesis and Activation

Numerous studies document the role of CKs as key regulators of nodule organogenesis (Gonzalez-Rizzo et al., 2006; Murray et al., 2007; Tirichine et al., 2007) and IT formation/progression (Held et al., 2014; Jardinaud et al., 2016). Recent transcriptomic data show that expression of CK biosynthesis genes such as *isopentenyl transferases (IPT)* and *CYP735A* that contribute to the biosynthesis of the bioactive CK form trans-zeatin, as well as CK activation genes such as *LONELY GUYs* (LOGs), can be induced by a 3 h NF treatment (van Zeijl et al., 2015b). Moreover, *MtIPT1* and *MtCYP735A1* regulation is independent of the CK receptor *MtCRE1*, suggesting a direct (and possibly local) production of CK as an early event following NF signaling. Similarly, *LjIPT2* and *LjLOG4* are regulated by NF application independently of the CK receptor *LjLHK1* in *L. japonicus* (Reid et al., 2017). Accumulation of bioactive CKs is also detected in the root susceptible zone following a 3 h NF treatment, and this is dependent on the NF signaling gene *MtDMI3* (van Zeijl et al., 2015b). Several genes from the trans-zeatin metabolic network are regulated in RHs after a 24 h NF treatment, suggesting accumulation of the bioactive CK trans-zeatin upon NF treatment. However, bioactive CK levels have not been measured in NF treated RHs (Jardinaud et al., 2016).

#### Inactivation and Degradation

Interestingly, CK inactivation enzymes adenyl phosphoribosides transferases (APT) and cytokinin oxidases (CKX) are also induced by a 3 h NF treatment but in an *MtCRE1* dependent manner, suggesting downstream negative regulatory feedback loops on CK accumulation (see **Figure 1**). Indeed, expression of the *AtCKX3* gene in *M. truncatula* plants under an epidermis specific promoter enhanced the number of ITs and nodules formed upon rhizobium inoculation. In contrast, expression of *AtCKX3* under a cortical specific promoter reduced nodulation (Jardinaud et al., 2016). In *L. japonicus*, NFs also induce expression of the *LjCKX3* gene but its promoter activity is specific to inner root tissues. Inactivation of *LjCKX3* leads to enhanced production of bioactive CK forms and reduces nodule organogenesis and IT formation (Reid et al., 2016). Altogether, this suggests that reducing the levels of active CKs may increase the efficiency of the infection process. This fits with the model suggested by Held et al. (2014) that argues for an inhibitory feedback loop driven by CK accumulation in the epidermis at late stages of nodulation. This is also consistent with antagonistic effects of CK application on the induction of the NF responsive gene *MtENOD11*. Indeed, a 3 h CK pretreatment of *M. truncatula* roots significantly reduces the induction of *MtENOD11* by NF. Conversely, epidermal expression of the *AtCKX3* gene enhanced the NF induction of *MtENOD11* (Jardinaud et al., 2016). In summary, growing evidence suggests that NFs induce CK production, which first controls nodule organogenesis, then rapidly activates negative feedbacks on NF signaling and infection processes, notably in the epidermis.

### Gibberellins

Increasing evidence also suggests a role for gibberellins (GAs) in controlling early events of symbiosis. A 24 h NF treatment induces both GA metabolic and biosynthetic genes in *M. truncatula* RHs, suggesting accumulation of the bio-active GA precursor GA12 in the root epidermis upon NF treatment (Breakspear et al., 2014; Jardinaud et al., 2016). An important NF-dependent and transient activation of GA biosynthesis genes was observed in soybean roots 12 hpi with rhizobia (Hayashi et al., 2012). Interestingly, 10^-7^ M GA3 treatment was previously shown to suppress NF-induced expression of the transcription factors *NSP2* and *NIN* in *L. japonicus* and 10^-6^ M GA3 suppressed NF induced RH deformation (Maekawa et al., 2009). Likewise, pretreatment of *M. truncatula* roots with 10^-6^ M GA3 suppressed NF dependent *ENOD11* induction. NF treatment also induced expression of the GA catabolic and biosynthetic enzymes GA2 and GA20ox, respectively (Fonouni-Farde et al., 2016). Moreover, this GA2ox catabolic gene was positively regulated by a 10 h NF treatment (Herrbach et al., 2017) and down-regulated in RHs after a 24 h NF treatment (Zaat et al., 1989; Breakspear et al., 2014). These data suggest that NFs can rapidly induce the biosynthesis of bioactive GA in RHs, which consequently triggers a negative feedback leading both to the downregulation of NF signaling and the activation of GA catabolic enzymes (see **Figure 1**). Increasing evidence also shows that bioactive GAs negatively regulate nodulation and infection in both determinate and indeterminate nodules (Maekawa et al., 2009; Fonouni-Farde et al., 2016). Thus, NF induced GAs could help fine-tune NF signaling and rhizobium infection during symbiosis.

### Ethylene

Previous genetic, physiologic, and pharmacologic studies highlight the key regulatory role ethylene plays in early symbiotic processes (Zaat et al., 1989; Heidstra et al., 1997; Oldroyd et al., 2001; Penmetsa et al., 2008). NFs can induce the expression of several ethylene biosynthetic genes, including two 1-aminocyclopropane-1-carboxylate (ACC) synthase genes (*MtACS1* and *MtACS2*; van Zeijl et al., 2015b). Moreover, three *ACS* genes are upregulated by NF signaling at 6 hpi with rhizobium (Larrainzar et al., 2015). Similarly, NF dependent ethylene production is detected in *L. japonicus* roots as early as 6 hpi (Reid et al., 2018). *MtACS3* is also synergistically regulated by a 10 h treatment with a combination of NFs and auxin (Herrbach et al., 2017). Promoter:GUS fusions showed RH expression of three *ACS* genes in *M. truncatula* (Larrainzar et al., 2015). This is consistent with previous data obtained in *L. japonicus* where a 1 h treatment with 10^-8^ M NF increased the expression of the ACC oxidase *LjACO2*, which was synergistically regulated by the combined application of NFs and ACC (Miyata et al., 2013). In contrast, NF-dependent downregulation of ethylene biosynthesis was observed at 48 hpi in the susceptible zone of soybean roots (Hayashi et al., 2012). Altogether, these data suggest that NF signaling rapidly and transiently induces the production of a negative regulator of infection, ethylene.

### Auxin

#### Auxin Metabolism

Nod factor-dependent induction of genes encoding auxin biosynthetic enzymes such as *YUCCA* and *ANTHRANILATE SYNTHASE* (*ASA*) was observed early after *Sinorhizobium meliloti* inoculation in whole roots (Larrainzar et al., 2015). Several auxin signaling genes are activated in RHs after a 24 h NF treatment (Breakspear et al., 2014), which argues for an effect of NFs on auxin production and signaling (see section “Auxin Signaling”). Similarly, we recently described a synergistic effect of NFs and auxin on the transcription of a large number of hormone biosynthesis genes, including an auxin biosynthesis *TRYPTOPHAN AMINOTRANSFERASE*-related (*TAR2*) homolog (Herrbach et al., 2017). Interestingly, a *GH3.1* gene, predicted to encode an auxin conjugation and inactivation enzyme (Yang et al., 2015) is expressed both in NF treated RHs as well as developing nodules (Breakspear et al., 2014; Ng et al., 2015; Jardinaud et al., 2016). We found this same *GH3* gene to be synergistically upregulated by NFs and auxin (Herrbach et al., 2017). This suggests fine tuning of bioactive pools of auxin, in RHs and probably inner cortical tissues for indeterminate nodules, upon NF perception and rhizobium infection.

#### Auxin Transport

Several studies link NF signaling to auxin transport. Spot inoculation of compatible rhizobia (but not a non-host strain) or micro-targeting of specific LCOs can modify auxin gradients, as measured by the GH3:GUS reporter gene in white clover (Mathesius et al., 1998). Likewise, flavonoid application also inhibits auxin transport. NF application or *S. meliloti* infection inhibits auxin transport from shoot to root at 24 h, as well as regulating expression of some *MtPIN* auxin efflux transporter genes, in an *MtCRE1* dependent manner (van Noorden et al., 2006; Plet et al., 2011; Ng et al., 2015). Moreover, MtCRE1 dependent pathways also control the accumulation of flavonoids in *M. truncatula* roots upon infection and flavonoid application can rescue the *Mtcre1* nodulation phenotype. These data suggest that NF induced, CK signaling triggers flavonoid induction and the subsequent inhibition of polar auxin transport. The resulting accumulation of auxin initiates cortical cell division and nodule organogenesis (**Figure 1**).

### Strigolactones

Strigolactones (SGLs) show a dose-dependent effect on nodulation in *M. truncatula* (Gomez-Roldan et al., 2008; De Cuyper et al., 2015). Interestingly, several studies (Larrainzar et al., 2015; van Zeijl et al., 2015a; Herrbach et al., 2017) showed a direct NF regulation of the expression of the SGL biosynthesis gene *DWARF27* (D27). The promoter of the *D27* gene is active in the root epidermis after a 3 h NF treatment and upon early steps of nodule organogenesis (van Zeijl et al., 2015a). Moreover, we observed that a combined auxin+NF treatment reduced the NF induction of *D27* expression, suggesting that auxin can antagonize NF effects (Herrbach et al., 2017).

### Jasmonic Acid (JA) and Brassinosteroids (BR)

Jasmonic acid (JA) and BR regulate plant stress responses and plant growth, but their role in RL symbiosis is not well understood. Conflicting reports indicate that host responses to these hormones vary depending on the legume species, hormone dose, or type of treatment studied (see Ferguson and Mathesius, 2014 for review and Conclusion and Perspectives section below). Some evidence suggests NF regulation of JA biosynthetic enzymes. Larrainzar et al. (2015) observed NF-dependent induction of two JA biosynthesis genes at 3–6 hpi and Breakspear et al. (2014) observed downregulation of a few JA biosynthetic genes in RHs after a 24 h NF treatment. In contrast, NF-dependent upregulation of several JA biosynthesis genes was observed in soybean at 48 hpi, while there seemed to be a reduction in expression of BR biosynthetic genes (Hayashi et al., 2012).

Hence, these data suggest that NF-signaling regulates several hormonal biosynthesis and activation pathways, with subsequent negative feedback loops that are rapidly activated after a brief NF treatment or upon rhizobium inoculation. Further evidence for a temporal regulation of bioactive hormone pools by NF signaling comes from the downstream regulation of many hormonal signaling genes (**Table 1**).

## NF Regulation of Hormone Signaling Genes

### CK Signaling

CK signaling utilizes a two-component signaling system comprised of a histidine-kinase (HK) receptor, phospho-transfer proteins (AHPs), and response regulators (RRs). Two types of RRs operate in CK signal transduction: A-type RRs are transcriptionally regulated upon CK perception but do not contain a DNA binding domain, and are generally considered as negative regulators of the CK response. In contrast, B-type RRs contain a Myb DNA binding domain, function as transcription factors and are generally considered as positive regulators (Kieber and Schaller, 2014). In *M. truncatula* and *L. japonicus*, the HK genes *MtCRE1* and *LjLHK1* have positive roles in cortical cell divisions and nodule organogenesis (Gonzalez-Rizzo et al., 2006; Murray et al., 2007; Tirichine et al., 2007). Recent transcriptomic and promoter fusion analyses demonstrate the induction of these CK receptor genes upon rhizobium infection or NF application; in Lotus, this epidermal expression inhibits infection (Held et al., 2014; Jardinaud et al., 2016).

Independent data suggest NF induced, RR gene expression occurs downstream of CK receptor activation. For instance, the A-type *MtRR4*, *MtRR8*, *MtRR9*, *MtRR11*, and the B-type *MtRR1* are expressed in *M. truncatula* roots following inoculation with either NF or wild-type *S. meliloti*, but not with nod^-^ *S. meliloti* mutants (Gonzalez-Rizzo et al., 2006; Op den Camp et al., 2011; van Zeijl et al., 2015b; Jardinaud et al., 2016). Likewise, an HK and an RR (*GmRR5*) gene are also induced in soybean in an NF-dependent manner (Hayashi et al., 2012). Finally, the CK signaling reporter construct, TCSn:GUS, can be induced in *M. truncatula* RHs upon a 4 h NF treatment (Jardinaud et al., 2016).

Altogether, an early activation of CK signaling probably occurs both in RHs and cortical cells, directly downstream of the NF signaling pathway (**Figure 1**).

### Auxin Signaling

Accumulating evidence suggests that NFs regulate the expression of auxin signaling genes. NF application induces the auxin related transcription factors genes *MtARF16a* and *MtPLETHORA3* in RHs, while several *ARF* genes are downregulated in RHs after a 24 h NF treatment (Breakspear et al., 2014; Jardinaud et al., 2016). These genes are also induced by auxin in *M. truncatula* roots (Herrbach et al., 2017). This suggests that NF perception could lead to auxin accumulation that would in turn activate some specific auxin signaling genes (**Figure 1**). Such genes could control cell divisions or IT formation (Breakspear et al., 2014).

### GA Signaling

DELLAs are well-known negative regulators of GA signaling (Sun, 2011). Recent data showed a positive effect of DELLAs on nodulation, and more specifically on IT formation (Fonouni-Farde et al., 2016; Jin et al., 2016). DELLAs reside in the NSP1/NSP2/IPD3 transcription factor complex that mediates NF signaling (Fonouni-Farde et al., 2016; Jin et al., 2016). External application of GAs can repress NF induction of several genes, such as *ERN1*, *ENOD11*, *NSP2*, and *NIN* (Maekawa et al., 2009; Fonouni-Farde et al., 2016). However, NFs do not induce DELLA gene expression (Fonouni-Farde et al., 2016).

### Ethylene and JA Signaling

Larrainzar et al. (2015) observed a biphasic regulation of expression of 47 AP2/ERF transcription factor genes in *M. truncatula* upon *S. meliloti* infection. The first wave of AP2/ERF expression takes place 1 hpi independent of NF perception, whereas the second wave of NF dependent, ethylene-regulated transcription occurs 6 hpi (Larrainzar et al., 2015). Although little is known about JA signaling during symbiosis, a 24 h NF treatment downregulates the expression of the putative JA receptor *COI1* in *M. truncatula* RHs implying that NF perception may attenuate this signaling pathway (Breakspear et al., 2014).

## Conclusion and Perspectives

Growing evidence shows that early events of NF signaling lead to the production and/or activation of hormones such as CK and GAs and the activation of their downstream signaling pathways. These hormones, in turn, interfere with other hormonal pathways, such as ethylene, ABA, and auxin, which can impinge upon NF signaling itself. For example, external application of CK or GA inhibits NF induction of *MtENOD11* (Fonouni-Farde et al., 2016; Jardinaud et al., 2016). Similarly, JA, ABA, and ethylene are well-known negative regulators of NF signaling (Oldroyd et al., 2001; Sun et al., 2006; Ding et al., 2008). We observed that auxin both positively and negatively regulated different NF responsive genes in a combined auxin + NF treatment (Herrbach et al., 2017) providing further evidence for the regulation of symbiosis signaling pathways by hormones. These negative effects may underpin the mechanism by which the host controls nodulation following the initial NF/rhizobium recognition.

It should, however, be noted that the concentrations of hormones applied in these studies are often in the micro-molar range. In the absence of quantitative data reporting endogenous hormone levels during symbiotic responses, it is difficult to assess if these studies reflect biologically relevant responses to plant hormones and this field requires such analyses.

Furthermore, recent evidence suggests that hormones function in a tissue-specific manner during symbiotic responses (see Gamas et al., 2017) but the specificity of these hormonal pathways is only partly resolved. Future work should address the respective contribution of different root tissues in producing, sensing, and responding to NF induced hormones, using tissue specific tools or quantitative hormone biosensors (Jones et al., 2014; Liao et al., 2015).

Finally, the NF signaling pathway overlaps with other symbiotic and developmental pathways suggesting that hormones may also impinge upon these processes. Similar to Rhizobia, arbuscular mycorrhizal fungi produce LCOs and utilize the same core signaling pathway to initiate symbiosis (Maillet et al., 2011). Future work should address whether LCO/hormone crosstalk regulates mycorrhization, where evidence already implicates a role for auxin, SGLs, GA, and LCO pathways (Lauressergues et al., 2012; Floss et al., 2013; Etemadi et al., 2014). Likewise, LCOs stimulate LR development and some NF induced genes are also involved in LR development (Herrbach et al., 2017). How LCO/hormone crosstalk could be involved in LCO stimulation of lateral root formation and how this could compare to its role in nodulation still requires further investigation.

## Author Contributions

SB and WB drafted the review and produced **Figure 1**. SB wrote the review with help of WB. WB produced **Table 1**.

## Conflict of Interest Statement

The authors declare that the research was conducted in the absence of any commercial or financial relationships that could be construed as a potential conflict of interest.
